# Cytotoxic effect of *Montivipera bornmuelleri’s* venom on cancer cell lines: in vitro and in vivo studies

**DOI:** 10.7717/peerj.9909

**Published:** 2020-10-27

**Authors:** Carol Haddoub, Mohamad Rima, Sandrine Heurtebise, Myriam Lawand, Dania Jundi, Riyad Sadek, Sebastian Amigorena, Ziad Fajloun, Marc C. Karam

**Affiliations:** 1 Department of Biology, University of Balamand, Kalhat, Al-Kurah, Lebanon; 2 Institut de Génétique et de Biologie Moléculaire et Cellulaire (IGBMC), INSERM, CNRS, University of Strasbourg, Strasbourg, France; 3 Institut Curie, INSERM U932, PSL Research University, Paris, France; 4 LAB3B, Doctoral School for Sciences and Technology, Azm Centre for Research in Biotechnology, Lebanese University, Tripoli, Lebanon; 5 Department of Neuroscience, Institute of Biology Paris-Seine (IBPS), INSERM, CNRS, Université Sorbonne-Nouvelle (Paris III), Paris, France; 6 Department of Biology, American University of Beirut, Beirut, Lebanon; 7 Faculty of Sciences 3, Michel Slayman Tripoli Campus, Lebanese University, Tripoli, Lebanon

**Keywords:** *Montivipera bornmuelleri*, B16 cells, MCA cells, Anti-cancer activity, In vivo

## Abstract

**Background:**

*Montivipera bornmuelleri*’s venom has shown immunomodulation of cytokines release in mice and selective cytotoxicity on cancer cells in a dose-dependent manner, highlighting an anticancer potential. Here, we extend these findings by elucidating the sensitivity of murine B16 skin melanoma and 3-MCA-induced murine fibrosarcoma cell lines to *M. bornmuelleri*’s venom and its effect on tumor growth in vivo.

**Methods:**

The toxicity of the venom on B16 and MCA cells was assessed using flow cytometry and xCELLigence assays. For in vivo testing, tumor growth was followed in mice after intratumoral venom injection.

**Results:**

The venom toxicity showed a dose-dependent cell death on both B16 and MCA cells. Interestingly, overexpression of ovalbumin increased the sensitivity of the cells to the venom. However, the venom was not able to eradicate induced-tumor growth when injected at 100 µg/kg. Our study demonstrates a cytotoxic effect of *M. bornmuelleri*’s venom in vitro which, however, does not translate to an anticancer action in vivo.

## Introduction

Cancer is characterized by an unregulated cell growth and division caused by the accumulation of mutations in genes that control cell proliferation/apoptosis and result in abnormal mass of tissue, called tumor. Tumors are classified as benign (not cancer) that does not invade nearby tissue, or as malignant (cancer), which can spread through the circulatory and lymphatic systems to invade other tissues and allow the growth of secondary tumors, or metastasis (Cooper & Hausman, 2004). Anticancer immunotherapy is achieved through several methods, including (i) monoclonal antibodies that are designed to mark cancer cells for elimination by binding to specific targets on their surface, (ii) cytokines including interferons and interleukins that enhance the immune response, (iii) Bacillus Calmette-Guérin (BCG) that treats bladder cancer by introducing a weakened form of tuberculosis bacteria to the bladder and inducing an immune response (Dillman, 1989), (iv) checkpoint inhibitors drugs that remove the breaks inhibiting T-Lymphocytes attack to cancer cells, (v) adoptive cellular therapy that uses an ex vivo manipulation of T-cells to induce an antitumor immune response, and (vi) vaccines designed specifically to boost the immune system against the patient’s tumor cells (McCune, 2016).

Cytokines are proteins secreted by cells for cellular communication (Zhang & An, 2007), serving as signals for growth, differentiation, and activation of immune cells (Dranoff, 2004). Cytokines enhance the activation of the effector and stromal cells of the immune system and the recognition of cancer cells at the tumor site. The Food and Drug Administration (FDA) has approved Interleukin 2 (IL-2) and Interferon-α (IFN-α) for cancer treatment. In fact, recent studies have shown that these two proteins are effective in treating skin cancer (melanoma) and renal cancer (Renal cell carcinoma) (Lee & Margolin, 2011; Sukari et al., 2016). However, the acquired resistance to cancer immunotherapy has pushed researchers to investigate new cancer treatment strategies, some of which are to use plant or algae extracts and animal venoms. Among animal venoms, snake venoms have been described as valuable source of new lead compounds in cancer treatment (Li, Huang & Lin, 2018).

*Montivipera bornmuelleri* is a venomous snake endemic to the high mountains of Lebanon found at elevations that exceed 1,900 m. It inhabits cold mountains, grasslands, rocks, and cushion-type vegetation (Hraoui-Bloquet et al., 2012). Many studies helped in the characterization of *M. bornmuelleri*’s venom by providing a detailed description of the venom content and its biological activities (reviewed in Rima et al. (2018)). Recently, it has been shown that *M. bornmuelleri*’s venom is selectively cytotoxic on keratinocyte cancer cell lines having different degrees of malignancy. In fact, *M. bornmuelleri*’s venom exerts greater cytotoxicity on the benign A5 and the low-grade malignant II4 cells than the non-tumorigenic HaCaT cell line, which correlates with their malignancy (Sawan et al., 2017). This study was the first to highlight the potential anticancer effects of this venom. Later on, another study investigated *M. bornmuelleri*’s venom effect on pro-inflammatory cytokines in vivo. Interestingly, while pro-inflammatory and anti-tumor cytokines (INF-γ, TNF-α, IL-1β, IL-4, IL-17) were up-regulated, anti-inflammatory cytokine (IL-10) was down-regulated in mice injected with the venom, suggesting an immunomodulatory effect of the venom (Yacoub et al., 2018). These findings suggest an anticancer potential of *M. bornmuelleri*’s venom that is worth further investigation. Therefore, the aim of this study is to pursue the characterization of *M. bornmuelleri*’s venom anticancer activity by assessing its cytotoxicity on different murine cancer cell lines, in vitro, and to describe whether the observed toxicity on cancer cell lines can be translated to an inhibition of tumor growth in vivo.

## Materials and Methods

### Venom

Lyophilized venom was supplied by Dr. Riyad Sadek (American University of Beirut) and stored at −20 °C. Venom was dissolved in PBS prior to the experiment as stock solution of 1 mg/mL.

### Cell lines and culture

B16 skin melanoma cells and 3-MCA-induced murine fibrosarcoma cell lines used in this study were cultured in the Curie Institute. Each of these cell lines exists as a subclone overexpressing the ovalbumin protein: B16-OVA and MCA-OVA (Ehst, Ingulli & Jenkins, 2003). These lines were used in this study along with their respective controls: B16 cell line and MCA-Mock that was transfected by a control plasmid. B16 and MCA cell lines were maintained in culture medium consisted of RPMI 1640 Medium, GlutaMAX™ supplemented with 10% SVF and 1% Penicillin/Streptomycin (Gibco, Carlsbad, CA, USA). B16 culture medium was supplemented with 2 mg/ml Geneticin and 60 μg/ml Hygromycin. For MCA lines, 1 mg/ml Hygromycin was added to the culture medium. Culture flasks were maintained in a humidified incubator (5% CO_2_) at 37 °C and cell passaging was performed at 70–80% of confluency. Passages were performed using the Trypsin acknowledged treatment. Briefly, the old medium was discarded and cells were washed with PBS. Then, cells were incubated with Trypsin for 5 min at 37 °C, inactivated with culture medium and transferred to falcon tubes. Cells were then centrifuged for 2 min at 1,000 rpm at RT, the supernatant was discarded and the pellet was resuspended in fresh medium. Cells were counted using the Dual Florescence LUNA™ Cell Counter and seeded at the desired concentration.

### EndoLISA assay

The detection of endotoxins in *M. bornmuelleri*’s venom was performed using ELISA-based Endotoxin Detection Assay (EndoLISA^®^) in accordance with the manufacturer’s instructions. First, a stock solution of Control Standard Endotoxin (CSE) was prepared at 500 EU/ml in endotoxin free water. Then, five CSE solutions of 50 EU/ml, 5 EU/ml, 0.5 EU/ml, 0.05 EU/ml, and 0.005 EU/ml were prepared by serial dilution. Five venom solutions were prepared (1 mg/mL, 10^−1^ mg/mL, 10^−2^ mg/mL, 10^−3^ mg/mL, and 10^−4^ mg/mL) by serial dilution in endotoxin free water. A total of 100 µL of each CSE/venom solution were pipetted in EndoLISA^®^ 96 wells plate. A total of 20 µL of the binding buffer was added to each well and the wells were sealed with a cover foil and incubated at RT for 18 h on a shaker (300 rpm). The liquid was then poured out rapidly in the sink, the plate was dried on a tissue paper, and each well was washed twice using the provided washing buffer (150 µL/well). A total of 100 µL of the detection solution was added to each well and the fluorescence was measured in a fluorescence microplate reader at T0 and after incubating the plate for 90 min at 37 °C. Fluorescence values were quantified using the formula: Fluorescence (F) = Fluorescence at T (F_T_) − Fluorescence at T0 (F_T0_)

### Cell viability measurement

Cells were seeded at a density of 1.25 × 10^4^ cell/well in a 24 well plate and allowed to adhere overnight at 37 °C, 5% CO_2_. The next day, the venom was added to the cells at different concentrations (20 μg/mL, 6.6 μg/mL, 2.2 μg/mL, 0.75 μg/mL and 0.25 μg/mL) and incubated for 24 and 48 h. Note that cells were checked under the microscope for any possible detachment; however, no detached viable cells were reported. At each desired time point, cells were harvested via the Trypsin acknowledged treatment. Briefly, the old medium was discarded and cells were washed with PBS. Then, cells were incubated with 100 µL of Trypsin for 5 min at 37 °C, detached by pipetting 100 µL of culture medium and transferred into 96 wells plate and centrifuged for 2 min at 1,000 rpm at RT. The supernatant was then discarded and the pellet was resuspended in 200 μL of Opti-MEM with DAPI (1:1,000), allowing the counting of viable cells using the MACS QUANT Flow cytometer.

### xCELLigence

Real-time monitoring of cell viability was performed using xCELLigence technology. To this end, 50 μL of medium was added in each well of the xCELLigence 16 well plate and incubated at 37 °C for 80 min. Afterwards, the old medium was discarded and replaced by 100 μL of fresh medium with the required concentration of suspended cells (10^5^ cells/ml for the B16 line and 2 × 10^5^ cells/ml for the MCA-Mock line). The plates were then incubated for 24 h in the xCELLigence at 37 °C, 5% CO_2_. On the next day, different venom solutions were prepared in culture medium and 100 μL of each venom solution was added per well to the preexisting medium to get a final venom concentration of 60 μg/mL, 20 μg/mL, 6.6 μg/mL, 2.2 μg/mL, 0.75 μg/mL and 0.25 μg/mL. The plates were then incubated at 37 °C on the xCELLigence. Cell viability was monitored over 72 h and represented as the unitless parameter cell index.

### Mice handling, ethics, and venom injection

C57BL/6 mice obtained from Charles River Laboratories were fed a standard diet and kept at 25 °C in 12 h day/night cycle. Animal care and use for this study were performed in accordance with the recommendations of the European Community (2010/63/UE) for the care and use of laboratory animals. Experimental procedures were specifically approved by the ethics committee of the Curie Institute CEEA-IC #118 (CEEA-IC 2014-03 and CEEA-IC 2017-006) in compliance with the international guidelines. Euthanasia was performed using gradual increase in CO_2_ concentration or cervical dislocation. All mice were sacrificed at the end of the experiment.

For physiological observations, mice were divided into 4 groups and injected subcutaneously with different doses of the venom: 100 µg/kg, 30 µg/kg, 10 µg/kg, 3 µg/kg. Each mice underwent 3 injections with a time interval of 3 days. Control animals were injected with PBS. After the first injection, the weight and skin condition of the mice were reported daily for 2 weeks to detect possible health problem, necrosis or hemorrhage.

### Tumor induction and size measurement

Mice were subcutaneously injected on both the right and the left abdomen sides with 5 × 10^5^ B16-OVA melanoma cells, and the tumor was allowed to grow for 8 days. Afterwards, the venom was injected at 100 µg/Kg only in the right tumor of the mouse. Mice injected with PBS and ADU-S100 (50 µg/Kg) were used as negative and positive controls, respectively. Each mice underwent 3 injections with a time interval of 3 days. The tumor length and width were measured every 3 days and the tumor volume was calculated accordingly as described in Kim et al. (2015). Once the tumor size has exceeded the volume of 2,000 mm^3^, the mice were sacrificed according to the Institutional Animal Care and Use Committee guidelines.

### Statistical analysis

Differences among groups were analyzed with GraphPad Prism 6.0 software (GraphPad Software Inc., San Diego, CA, USA) using Student’s *t*-test for in vitro experiments and one-way ANOVA followed by Tukey’s multiple comparison test was performed for in vivo experiments. Results were expressed as means ± SD unless mentioned differently.

## Results

### *Montivipera bornmuelleri*’s venom content of endotoxin

**Figure 1:**
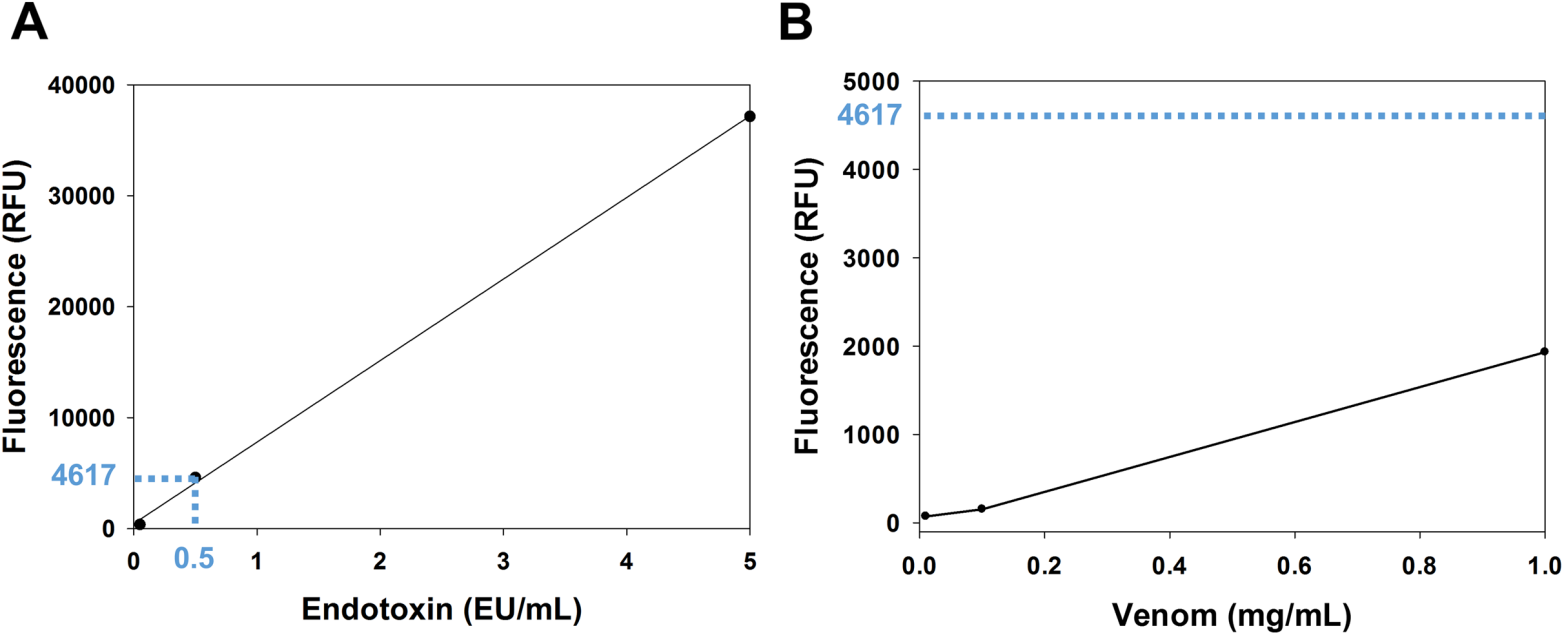
Analysis of venom content of endotoxin using EndoLISA assay. (A) Endotoxin standard curve showing (in blue) the fluorescence value of the accepted limit of endotoxin (0.5 EU/mL). (B) Venom fluorescence values assessed by EndoLISA. The blue dashed line represents the fluorescence (4617 RFU) of the accepted limit of endotoxin according to the U.S. Food and Drug Administration.

The venom content of endotoxin was determined using the EndoLISA assay. The comparison between the venom fluorescence curve and the accepted limit of endotoxin shows that the endotoxin content of the venom is whithin the acceptable range. In fact, according to the U.S. Food and Drug Administration, the accepted limit of endotoxin is 0.5 EU/mL (Gorbet & Sefton, 2005). In the endotoxin standard curve (Fig. 1A), this value corresponds to a fluorescence of 4,617 RFU (relative fluorescence unit), which is ~2.4 fold higher than the fluorescence of the venom (1,932 RFU) at the highest concentration used (1 mg/mL) (Fig. 1B).

### Cytotoxicity of *M. bornmuelleri*’s venom on B16 and MCA cell lines

**Figure 2:**
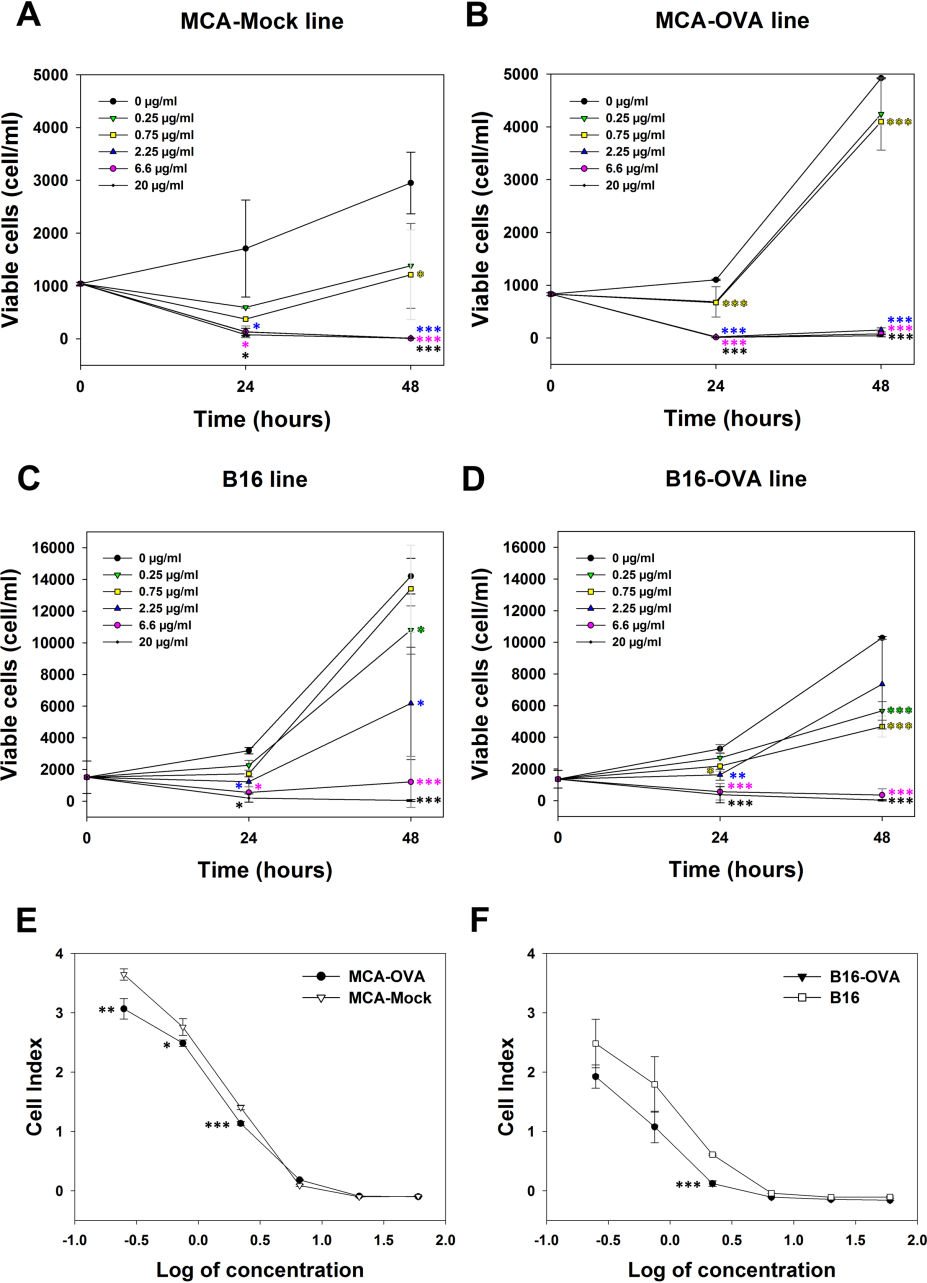
Cytotoxicity of *M. bornmuelleri*’s venom on B16 and MCA cell lines. Quantification of (A and B) MCA and (C and D) B16 viable cells, 24 and 48 h post exposure to different *M. bornmuelleri*’s venom concentrations. Significance of each group is statistically determined as compared to the control and color-coded based on the concentration color-code. (E and F) Real-time analysis of MCA and B16 cell viability over 72 hrs in presence of different *M. bornmuelleri’s* venom concentrations. Cell viability is represented as the unitless parameter cell index. *p < 0.05, **p ≤ 0.01 and ***p ≤ 0.001.

The cytotoxicity of *M. bornmuelleri*’s venom against cancer cell lines was assessed on B16 skin melanoma cells and 3-MCA-induced murine fibrosarcoma cell lines by quantifying viable cells 24 and 48 h after venom addition. Results show a dose dependent cytotoxicity of the venom on the two cell lines (Fig. 2). At 24 h, *M. bornmuelleri*’s venom shows significant cytotoxic effect at doses equal to/or exceeding 2.25 μg/mL for MCA-Mock cells and 0.75 μg/mL for MCA-OVA cells (Figs. 2A and 2B), suggesting that ovalbumin expression increases cells sensitivity to the venom. Beyond 6.6 μg/mL, the venom kills all MCA-Mock and MCA-OVA cells (Figs. 2A and 2B). However, 20 μg/mL and 6.6 μg/mL of *M. bornmuelleri*’s venom are required to kill all B16 and B16-OVA cells, respectively, 48 h post-treatment (Figs. 2C and 2D). Note that OVA expression seems to affect MCA and B16 cell growth in an opposite way. While MCA-OCA cell proliferation looks accelerated, the proliferation of B16-OVA cells seems to be retarded compared to MCA-Mock and B16 cell lines, respectively (Figs. 2B and 2D).

To validate the cytotoxic activity of *M. bornmuelleri*’s venom, live cell proliferation was monitored for 72 h using xCELLigence. The venom effect on B16 and MCA cells was therefore analyzed by following the evolution of the cell index at different venom concentrations. The dose-dependent decrease of cell index shown in Fig. 2C validated the dose-dependent toxicity of *M. bornmuelleri*’s venom on the four tested cell lines. Interestingly, OVA-expressing cells showed lower cell index compared to their respective parental cells (Figs. 2E and 2F); suggesting that OVA-expressing cells are more sensitive to the venom. The half maximal inhibitory concentrations (IC_50_) were estimated at 0.79 µg/mL for B16 cells, 0.36 µg/mL for B16-OVA cells, 1.1 µg/mL for MCA-Mock cells, and 1.2 µg/mL for MCA-OVA cells. The B16 cell lines show lower IC_50_ values compared to those of MCA cell lines, and thus are more sensitive to the venom. B16-OVA cells, having the lowest IC_50_ among the four tested cell lines, were used for the in vivo experiments.

### Effects of *M. bornmuelleri*’s venom subcutaneous injections

**Figure 3:**
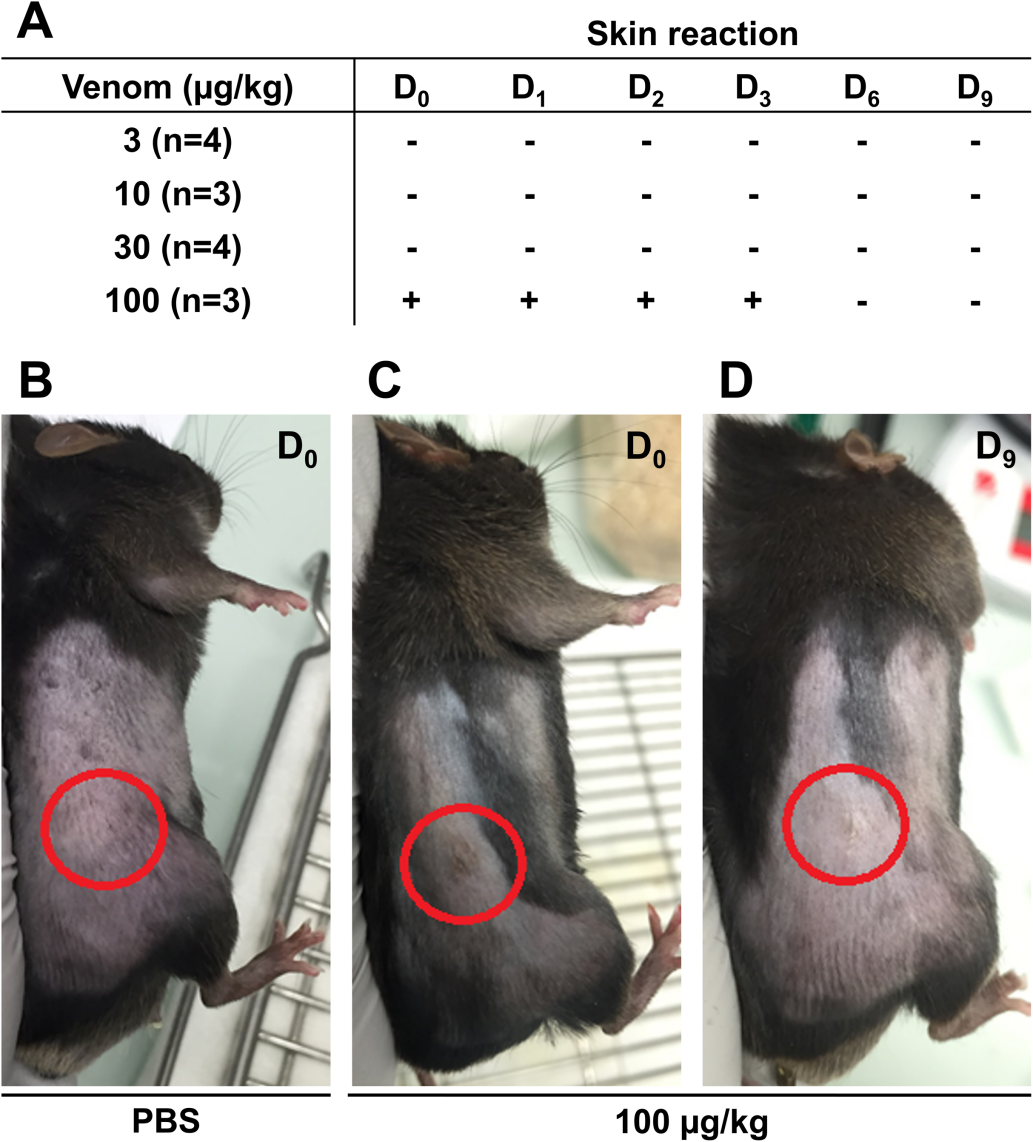
Skin condition after subcutaneous injection of C57Bl6/J mice with different venom concentrations. The venom was injected at *D*_0_ at the frequency of three injections with a time interval of 3 days and mice were checked for signs of inflammation over 9 days. (A) No skin reaction at the injection site was detected for venom doses of 3 µg/kg, 10 µg/kg, and 30 µg/kg from *D*_0_ to *D*_9_. However, compared to PBS (B), inflammation was detected at the injection site (circles) few hours post-injection (*D*_0_) (C), remained until day 3 (*D*_3_), and then was fully recovered (D).

Before starting the in vivo experiments, the optimal venom dosage to be injected subcutaneously in mice was determined. To this end, 14 male C57Bl6/J mice were injected subcutaneously with different venom doses and mice weight and skin condition at the injection site were reported daily to detect possible health problem. The weight of the mice was measured throughout the experiment to ensure they did not lose more than 20% of their weight, which is the acceptable limit according to the guidelines of the Institutional Animal Care. No weight loss exceeding the acceptable limit was reported even at the highest venom concentration (data not shown). Skin condition was monitored for 9 days to detect any inflammatory reaction, hemorrhage, or necrosis. Results presented in Fig. 3 show that the venom did not induce any sign of skin reaction at 3 µg/Kg 10 µg/Kg, and 30 µg/Kg (Fig. 3A). However, at 100 µg/Kg, mice showed inflammation at the injection site, after the first injection, that was recovered after 3 days (Figs. 3B–3D). After monitoring mice weight and skin condition, mice were dissected to check for internal organs damage. Results showed that mice internal organs were intact with no necrosis or hemorrhage detected at different injected venom concentrations (data not shown). Therefore, the venom concentration chosen to be used in the next experiment was 100 µg/Kg.

### Effects of *M. bornmuelleri*’s venom on tumor progression in vivo

**Figure 4:**
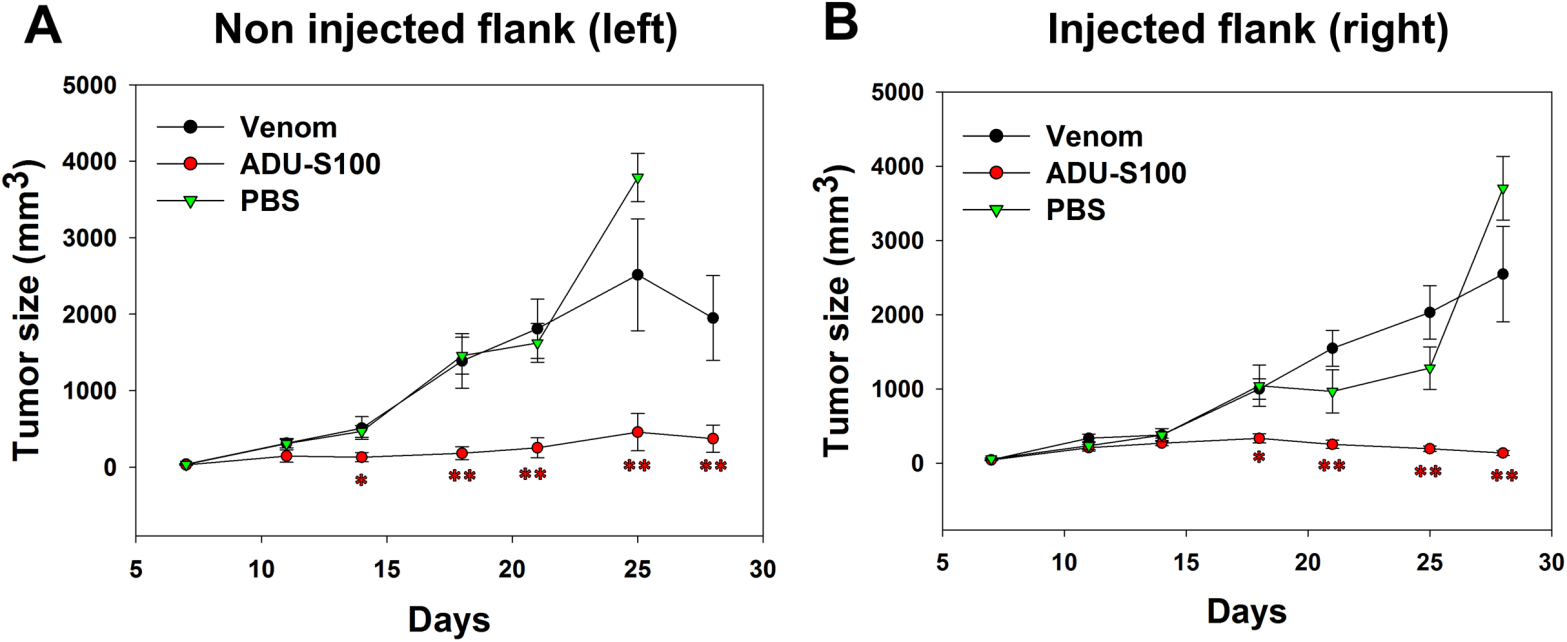
Quantification of tumor size in left (A) and right (B) flanks after intratumoral injections of *M. bornmuelleri*’s venom at the right side of mice abdomen. PBS and ADU-S100 were used as negative and positive controls, respectively. Results are represented as mean ± SEM. PBS-injected mice *n* = 10, ADU-S100-injected mice *n* = 12, venom-injected mice *n* = 12. *p < 0.05 and **p ≤ 0.01.

B16-OVA cells were injected subcutaneously on both sides of the mice abdomens and tumor was allowed to grow for 8 days. Intratumoral venom injections were performed only at the right side of the abdomen and tumor size was measured at both sides to check if the venom has any local or systemic effect on tumor growth. Contrarily to the positive control, the venom did not induce any significant reduction in the left tumor size that increased in a similar way to the tumor in PBS-treated mice, suggesting the absence of any systemic inhibition of tumor growth (Fig. 4A). Similarly, the right tumor kept gradually growing in venom-treated mice as in PBS-treated mice, suggesting that the venom is unable to induce any local inhibition of tumor progression (Fig. 4B). However, ADU-S100-treated mice show a significant decrease in both left and right tumor sizes, suggesting a local and systemic inhibition of tumor growth (Figs. 4A and 4B).

## Discussion

Different biological activities of *Montivipera bornmuelleri*’s venom have been recently described (for review, see Rima et al., 2018). Among these, the selective cytotoxicity of *M. bornmuelleri*’s venom on benign and malignant cells (Sawan et al., 2017) and its upregulation of pro-inflammatory cytokines (Yacoub et al., 2018) suggested an anticancer potential of the venom. To further validate this hypothesis, this study was conducted to dissect the cytotoxicity of *M. bornmuelleri*’s crude venom on cancer cell lines and its effect on tumor growth in vivo. We started our experimental approach by checking whether the impact of *M. bornmuelleri*’s venom on pro-inflammatory cytokines levels described previously (Yacoub et al., 2018) is due to endotoxins within the venom. In fact, endotoxins are found on the outer membrane of most gram-negative bacteria (Raetz, 1990) and are known as strong inducers of the immune system (Singh & Jiang, 2003). At the highest tested venom concentration (1 mg/ml), endotoxins levels were significantly less than the accepted limit of endotoxin according to the U.S. Food and Drug Administration (Gorbet & Sefton, 2005). Therefore, the immunotherapeutic potential of *M. bornmuelleri*’s venom can be explored with no doubt about the presence of endotoxins in the venom may interfere with the inflammatory immune responses of mice.

To continue in examining venom cytotoxicity on cancer cells, we quantified melanoma (B16) and Fibrosarcoma (MCA) cell viability in presence of different venom concentrations and we reported a dose-dependent cytotoxicity of the venom on the two cell lines. This is in agreement with previous findings reporting anticancer activity of crude snake venoms (Calderon et al., 2014; Debnath et al., 2007; Kerkkamp et al., 2018; Li, Huang & Lin, 2018). Interestingly, B16 cells were more sensitive to the venom, compared to MCA cells, and interestingly, an increased sensitivity to the venom was observed when these cell lines overexpressed ovalbumin. It could possible that ovalbumin expression induces changes in cell cycle progression that affect cell growth (Gitlin et al., 2015) and influence cell sensitivity to the venom. This hypothesis was investigated in Lewis lung carcinoma cell lines; however, the overexpression of ovalbumin did not impact the tumor proliferation (Lu et al., 2011). Our findings show that indeed ovalbumin expression affected MCA and B16 cell growth, which was increased in MCA-OVA, and decreased in B16-OVA cells, compared to their respective parental cell lines. It could be possible that ovalbumin expression slows down B16 cell cycle and accelerates that of MCA cells. If true, this may explain why B16 cells are more sensitive to the venom that is able to pursue its cytotoxic activity before cells invade the plates. The exact mechanism of action of *M. bornmuelleri*’s venom on cancer cells need further investigation. Whether venom compounds affect the cell cycle is also possible. For example, it was reported that snake venom (Bernardes-Oliveira et al., 2016), and more precisely Phospholipases A2 (Prinholato da Silva et al., 2015), can lead to cell cycle arrest at the G0/G1 phase. *M. bornumelleri*’s venom is known to contain Phospholipases A2 (Accary et al., 2015); therefore, it will not be surprising if the venom affects the cell cycle in a similar way to what was reported previously (Bernardes-Oliveira et al., 2016; Prinholato da Silva et al., 2015).

Venom compounds that are responsible of the reported cytotoxicity of *M. bornmuelleri*’s venom on cancer cell models can be revealed by further proteomic studies. In fact, many previous studies were able to describe snake venom components with immunotherapeutic and anti-cancer potential due to their specificity, stability, selectivity, and small size (Li, Huang & Lin, 2018; Ma, Mahadevappa & Kwok, 2017; Rima et al., 2018). For example, obtustatin obtained from *Vipera lebetina obtusa* snake venom inhibited angiogenesis and decreased tumor size in the Lewis lung synergic mouse model (Marcinkiewicz et al., 2003), and S-180 sarcoma-bearing mice model (Ghazaryan et al., 2019). Cardiotoxin III isolated from *Naja naja atra* venom (Bhaskaran et al., 1994) have been shown to induce cytochrome c release and the induction of the caspase pathway favoring apoptosis in cancer cells (Yang et al., 2005). Of interest is the finding reporting that crotamine, known as antitumoral peptide from Rattlesnakes, inhibits melanoma growth in vivo even when orally administered, which highlights the diversity of possible administration routes (Campeiro et al., 2018). Other studies synthesized venom-derived peptides and validated their capacity in reducing tumor size in different mice models. For example, vicrostatin, a disintegrin derived peptide, prolonged the survival of glioma-bearing mice (Swenson et al., 2018), while synthetic peptides derived from Phospholipases A2 showed antitumor effects in vitro and in vivo (Araya & Lomonte, 2007). In agreement, Phospholipases A2 from *Bothrops jararacussu* venom antitumor and antimetastatic effects on human breast cancer cells (De Vasconcelos Azevedo et al., 2019).

*M. bornmuelleri*’s venom was not studied for its anticancer potential in vivo. Therefore, we assessed whether the cytotoxicity of the venom observed on cancer cells in vitro can be translated into tumor growth inhibition in mice. First, we described physiological consequences of *M. bornmuelleri*’s venom subcutaneous injection in mice, which go along the characterization of the venom intramuscular and intraperitoneal injection effects published previously (Abi-Rizk et al., 2017). Interestingly, the venom, even at the highest tested concentration (100 µg/Kg), did not show any significant weight loss, nor induce hemorrhage, necrosis, internal organ damage, or lethality and was considered safe to use for mice subcutaneous injection. To check whether the venom is able to inhibit tumor cell progression in vivo as in vitro, subcutaneous induced tumor was injected with *M. bornmuelleri*’s venom and tumor size was followed for 3 weeks. Unfortunately, no significant reduction in tumor size was observed in venom-treated mice, suggesting an inability of the venom to limit tumor cells progression in the tested experimental conditions. Herein, different factors could be behind the negative result and should be kept in mind including cells growth speed, cell number, and venom dosage. It could be possible that the number of cells used for tumor induction is relatively high and, therefore, a high concentration of venom (higher than 100 µg/Kg) is needed to significantly affect tumor progression. In other words, the usage of crude venom, which is a mixture of enzymes and non-enzymatic proteins or peptides can impose some limitations. In fact, the available quantity of these compounds within a defined quantity of crude venom is limited and may therefore limit their effects, if present. Consequently, we emphasize the importance of venom fractionation that allows to concentrate purified venom compounds. This may reveal biological activities of interest that may not be detectable when using crude venom. In addition, B16 cells divide relatively fast, as observed by flow cytometry quantification, therefore, it could be possible that the venom toxicity is quenched by the fast proliferation of the cells. Also, cancer is far from being represented by cancer cell lines culture, where no connective tissue, extracellular matrix, and vascularization are present, as in animal models. That’s why venom’s effect may not yield the same response and may produce different and unexpected results in vivo. Therefore, further studies are still needed to check whether the venom cytotoxicity on cancer cell lines in vitro can be translated into tumor size regression in vivo.

## Conclusions

Our study stands with previous finding highlighting the anticancer potential of *M. bornmuelleri*’s venom in vitro (Sawan et al., 2017) and warrants further studies investigating its in vivo effect. It will be of interest to consider analytical studies to fractionate the venom and identify the compound(s) responsible of the cytotoxicity and their mechanism of action. Also, advanced studies are still needed to assess whether the cytotoxic effect observed in vitro can be reproduced in vivo. These studies may help in the identification and characterization of bioactive proteins of interest for new cancer therapeutic strategies.

## Supplemental Information

10.7717/peerj.9909/supp-1 Supplemental Information 1 Raw data for Figure 1.

10.7717/peerj.9909/supp-2 Supplemental Information 2 Raw data for Figure 2.

10.7717/peerj.9909/supp-3 Supplemental Information 3 Raw data for Figure 3.

10.7717/peerj.9909/supp-4 Supplemental Information 4 Raw data for Figure 4.

## Additional Information and Declarations

### Data Availability

The following information was supplied regarding data availability: Data are available as Supplemental Files.
